# Clinical relevance of pathogenic germline variants in mismatch repair genes in Chinese breast cancer patients

**DOI:** 10.1038/s41523-022-00417-x

**Published:** 2022-04-21

**Authors:** Li Hu, Jie Sun, Zhongwu Li, Ziwei Qu, Yan Liu, Qiting Wan, Jiaming Liu, Xinyun Ding, Fan Zang, Juan Zhang, Lu Yao, Ye Xu, Yin Wang, Yuntao Xie

**Affiliations:** 1 Familial & Hereditary Cancer Center, Key Laboratory of Carcinogenesis and Translational Research (Ministry of Education), Peking University Cancer Hospital & Institute, 100142 Beijing, P. R. China; 2 Department of Pathology, Key Laboratory of Carcinogenesis and Translational Research (Ministry of Education/Beijing), Peking University Cancer Hospital & Institute, 100142 Beijing, P. R. China; 3 Berry Oncology Corporation, 350200 Fujian, P. R. China

**Keywords:** Breast cancer, Cancer genetics, Cancer immunotherapy

## Abstract

The prevalence and clinical relevance of pathogenic germline variants in MMR genes have not been investigated in large series of breast cancers. In this study, we screened the germline variants in MMR genes in 8085 consecutive Chinese breast cancer patients, and investigated the MMR/PD-L1 protein expression and tumor mutation burden (TMB) of breast tumors from MMR variant carriers. We found that 15 of 8085 patients (0.19%) carried a pathogenic germline variant in MMR genes. Compared with non-carriers, MMR variant carriers might have worse recurrence-free survival (unadjusted hazard ratios [HR] = 2.70, 95% CI: 1.12–6.49, *P* = 0.027) and distant recurrence-free survival (unadjusted HR = 3.24, 95% CI: 1.45–7.22, *P* = 0.004). More importantly, some of the breast cancers from MMR carriers displayed MMR protein loss (5/13), TMB-high (2/10), and PD-L1 positive expression (9/13). This study showed that MMR variant carriers were rare in breast cancer. They might have worse survival and part of them might benefit from immunotherapy.

## Introduction

Pathogenic germline variants in four mismatch repair (MMR) genes (MLH1, MSH2, MSH6, and PMS2) have been found to result in hereditary nonpolyposis colorectal carcinoma (HNPCC, also called Lynch syndrome), which is an autosomal dominant inherited disease primarily associated with colorectal, endometrial, gastric, small intestinal, hepatobiliary, renal pelvic, and ureteral cancers^1^. Currently, colorectal cancers from MMR variant carriers are associated with poor differentiation, extensive lymphocytic infiltration and a superior survival compared with sporadic cases^2,3^. More importantly, disfunction of MMR genes in HNPCC results in MMR protein loss, and induces errors of DNA polymerase during DNA replication, causing the accumulation of mutations in the genome, especially in the repetitive DNA sequences called microsatellites^4^. As a consequence, HNPCCs typically exhibit loss of MMR protein expression, microsatellite instability (MSI) and high tumor mutation burden (TMB), which make them sensitive to immunotherapy^5,6^.

A recent clinical trial has highlighted the significant responses of solid cancers with MMR deficiency to immunotherapy, regardless of the cancer type^7^, which caused considerable interest in investigating MMR in various cancer types. Whether breast cancer can be recognized as part of the spectrum of HNPCC has been debated. A previous study concluded that breast cancer is not associated with HNPCC^8^. However, multiple studies reported that women from HNPCC families had an increased risk of breast cancer ranging from 2- to 4-fold compared with the general population^9–14^, and some of the breast cancers from HNPCC families exhibited MMR protein loss or MSI^15–21^. These results suggested a potential role for MMR deficiency in breast tumorigenesis. Notably, these previous studies focused on breast cancers from HNPCC families, while MMR genes have not been investigated in a large series of consecutive breast cancers.

In this study, we aimed to investigate the frequency of pathogenic germline variants in MMR genes (MLH1, MSH2, MSH6, and PMS2) in a large series of Chinese breast cancer patients. Then we compared the clinicopathological characteristics and survival between MMR variant carriers and non-carriers. Finally, we explored whether these breast cancer patients with germline MMR variants can potentially benefit from immunotherapy.

## Results

### Prevalence, clinical characteristics and survival of breast cancer patients carrying MMR germline variants

**Fig. 1:**
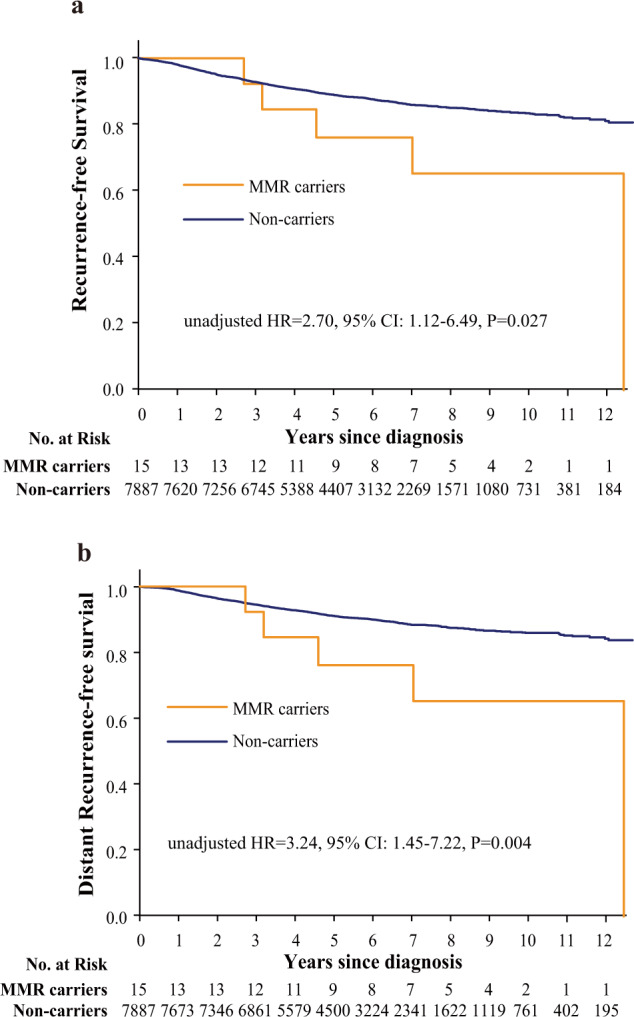
Kaplan–Meier survival analyses according to germline variants status in MMR genes. **a** and **b** show recurrence-free survival and distant recurrence-free survival, Data are analyzed for statistical significance using univariate Cox proportional hazards models. Two-sided *P* values < 0.05 were considered to be statistically significant.

A total of 15 patients in the 8085 Chinese consecutive breast cancer cohort (0.19%) carried a pathogenic germline variant in the four MMR genes, including PMS2 (*n* = 6), MSH6 (*n* = 5), MSH2 (*n* = 3), and MLH1 (*n* = 1) (Table 1). Six patients of the fifteen MMR variant carriers (40.0%) had a personal or family history of HNPCC-related cancers. Gastric cancer (*n* = 4) and colorectal cancer (*n* = 3) were the most common (Table 1). Compared with non-carriers, MMR variant carriers were more likely to have a positive family history of HNPCC-related cancer [40.0% (6/15) vs. 10.9% (882/8070), *p* = 0.001] (Table 2). No difference in age at diagnosis or family history of breast cancer was noted between the MMR variant carriers and non-carriers (Table 2). In terms of the tumor characteristics, most of the breast cancers from MMR variant carriers were invasive ductal carcinomas, while MMR variant carriers might have more medullary [6.7% (1/15) vs. 0.6% (47/8070), *p* = 0.08] and papillary (13.3% (2/15) vs. 0.3% (23/8070), *p* < 0.001] carcinomas compared with non-carriers (Table 2). There was no significant difference in tumor size, grade, lymph node metastasis, or ER/PR/HER2 status between the MMR variant carriers and non-carriers (Table 2). After the median follow-up time of 65.3 months, MMR variant carriers showed significantly worse recurrence-free survival (RFS) (unadjusted HR = 2.70, 95% CI: 1.12–6.49, *P* = 0.027) and distant recurrence-free survival (DRFS) (unadjusted HR = 3.24, 95% CI: 1.45–7.22, *P* = 0.004) than non-carriers (Fig. 1). After adjustment for age, tumor size, lymph node, tumor grade, ER/PR/HER2 status and treatment, pathogenic germline variants in MMR genes had a trend to be associated with worse RFS (adjusted HR = 2.34, 95% CI: 0.97–5.66, *P* = 0.06) and were significantly associated with worse DRFS (adjusted HR = 2.76, 95% CI: 1.14–6.69, *P* = 0.03) in the breast cancer patients in this study (Table 3).

**Table 1:** Fifteen breast cancer patients with pathogenic germline variants in MMR genes identified from 8085 breast cancer patients.

Case ID	Germline	Age (y)	Personal history	Family history	Histology	Tumor size	Lymph node	ER status	PR status	HER2 status
P12	PMS2:p.R134X	80	–	–	IDC	1.4*1.2	(−)	(−)	(−)	(−)
P14	PMS2:p.E225X	43	Lymphoma, 55 y	1-1 breast cancer, 42 y; 2-1 ovarian cancer, 27 y; 2-1 gastric cancer, 78 y	IDC	2.5*1.8	(+)	(+)	(+)	(−)
P20	PMS2:p.R315X	38	Cervical cancer, 39 y	–	IDC	2.1*2.0	(+)	(−)	(+)	(−)
P25	PMS2:p.L351X	45	–	1-1 gastric cancer, 45 y; 2-1 gastric cancer, 50 y; 2-1 esophagus cancer; 3-1 esophagus cancer	IDC	5.4*3.8	(+)	(+)	(+)	(−)
P26	PMS2:p.V754fs	34	–	–	IDC	4.6*1.3	(−)	(−)	(−)	(+)
P27	PMS2:p.R151fs	61	–	–	IDC	2.2*2.0	(−)	(+)	(+)	(−)
P5	MSH6:p.R495X	62	–	2-1 pancreatic cancer, 40 y; 2-1 liver cancer, 50 y	IDC	1.1*1.0	(−)	(+)	(+)	(−)
P8	MSH6:p.Y994X	50	–	1-1 breast cancer, 40 y	Invasive papillary	1.7*1.5	(+)	(+)	(+)	(−)
P22	MSH6:p.R922X	57	–	–	IDC	1.7*1.6	(+)	(−)	(−)	(+)
P23	MSH6:p.T716fs	67	–	–	IDC	1.4*1.4	(−)	(+)	(−)	(−)
P31	MSH6:p.T1085fs	53	Endometria cancer, 52 y	1-1 gastric cancer, 60 y; 2-1 gastric cancer, 71 y; 2-1 breast cancer, 50 y	Medullary	2.2*1.5	(−)	(−)	(−)	(−)
P2	MSH2:p.Q4X	54	–	2-1 colorectal cancer, 64 y	IDC	2.4*1.6	(+)	(+)	(+)	(−)
P19	MSH2:p.R877fs	64	–	1-1 brain cancer	IDC	1.6*1.7	(−)	NA	NA	NA
P21	MSH2:p.Q314fs	75	Rectal cancer, 77 y	–	Invasive papillary	2.5*2.2	(−)	(+)	(+)	(−)
P15	MLH1:p.R659X	46	Rectal cancer, 42 y	1-1 rectal cancer, 45 y; gastric cancer, 42 y; 1-1 lung cancer, 47 y	DCIS	2.0*1.8	(−)	(+)	(+)	(−)

*NA* not accessible, *DCIS* ductal carcinoma in situ, *IDC* invasive ductal carcinoma, *ILC* invasive lobular carcinoma, *MMR* mismatch repair, *ER* Estrogen receptor, *PR* progesterone receptor, *HER2* human epidermal growth factor receptor 2.

**Table 2:** Comparison of clinical characteristics between MMR variant carriers and non-carriers in a large cohort of breast cancer patients.

Characteristic	MMR carriers (*n* = 15)			Non-carrier (*n* = 8070)			*P* value
	*N*	%		*N*	%		
Age at diagnosis, years							
Mean ± SD	55.3 ± 13.2			51.1 ± 11.6			0.16
Early-onset							0.88
≤40	2	13.3		1466	18.2		
>40	13	86.7		6604	81.8		
Family history of HNPCC-related cancer							0.001
No	9	60.0		7188	89.1		
Yes	6	40.0		882	10.9		
Family history of breast cancer							0.38
No	12	80.0		7266	90.0		
Yes	3	20.0		804	10.0		
Family history of any cancers							0.39
No	8	53.3		5417	67.1		
Yes	7	46.7		2653	32.9		
Histology							<0.001
Ductal	12	80.0		7195	89.2		
Medullary	1	6.7		47	0.6		
Papillary	2	13.3		23	0.3		
Others	0	0.0		805	10.0		
Tumor size							0.44
≤2 cm	6	40.0		3283	40.7		
>2 cm	7	46.7		4392	54.4		
Unknown	2	13.3		395	4.9		
Tumor grade							0.86
I	0	0.0		625	7.7		
II	10	66.7		4599	57.0		
III	2	13.3		984	12.2		
Unknown	3	20.0		1862	23.1		
Lymph nodes status							0.41
Negative	9	60.0		5514	68.4		
Positive	6	40.0		2084	25.8		
Unknown	0	0.0		472	5.8		
ER status							0.68
Negative	4	26.6		2213	27.4		
Positive	10	66.7		5519	68.4		
Unknown	1	6.7		338	4.2		
PR status							0.84
Negative	4	26.7		2726	33.8		
Positive	10	66.7		4885	60.6		
Unknown	1	6.7		459	5.7		
HER2 status							0.83
Negative	12	80.0		5565	68.9		
Positive	2	13.3		1845	22.9		
Unknown	1	6.7		660	8.2		
Subtype							0.75
ER/PR + , HER2−	10	66.7		5733	71.1		
HER2 +	2	13.3		852	10.5		
ER−, PR−, HER2−	2	13.3		1103	13.7		
Unknown	1	6.7		382	4.7		

P, Carriers vs. Non-carrier.

*MMR* mismatch repair, *HNPCC* Hereditary nonpolyposis colorectal cancer, *ER* estrogen receptor, *PR* progesterone receptor, *HER2* Human epidermal growth factor receptor 2, *SD* standard deviation.

**Table 3:** Multivariate analyses of recurrence-free survival and distant recurrence-free survival in this cohort.

Variable	RFS			DRFS	
	HR (95% CI)	*P*		HR (95% CI)	*P*
Age					
≤40 yr	1.00	–		1.00	–
>40 yr	0.78 (0.66–0.94)	0.007		0.90 (0.73–1.10)	0.28
Tumor size					
≤2 cm	1.00	–		1.00	–
>2 cm	1.57 (1.32–1.86)	<0.001		1.73 (1.43–2.09)	<0.001
Lymph node					
Negative	1.00	–		1.00	–
Positive	3.41 (2.92–3.98)	<0.001		3.59 (3.03–4.25)	<0.001
Grade					
I	1.00	–		1.00	–
II	1.34 (0.99–1.82)	0.06		1.45 (1.03–2.04)	0.03
III	1.38 (0.97–1.98)	0.07		1.49 (1.00–2.21)	0.05
ER status					
Negative	1.00	–		1.00	–
Positive	0.73 (0.56–0.94)	0.01		0.77 (0.58–1.01)	0.06
PR status					
Negative	1.00	–		1.00	–
Positive	0.75 (0.60–0.94)	0.01		0.73 (0.57–0.92)	0.009
HER2 status					
Negative	1.00	–		1.00	–
Positive	1.13 (0.96–1.34)	0.15		1.18 (0.98–1.41)	0.08
Treatment					
No treatment	1.00	–		1.00	–
C vs. no treatment	1.09 (0.77–1.55)	0.63		0.94 (0.65–1.36)	0.74
E vs. no treatment	1.22 (0.86–1.73)	0.28		1.06 (0.73–1.53)	0.78
C + E vs. no treatment	1.03 (0.69–1.56)	0.87		0.88 (0.57–1.36)	0.56
Pathogenic variants					
Non-carriers	1.00	–		1.00	–
MMR carriers	2.34 (0.97–5.66)	0.06		2.76 (1.14–6.69)	0.03

*RFS* recurrence-free survival, *DRFS* distant recurrence-free survival, *CI* confidence interval, *C* chemotherapy, *E* endocrine therapy, *ER* estrogen receptor, *PR* progesterone receptor, *HER2* human epidermal growth factor receptor 2, *HR* hazard ratio, *MMR* mismatch repair.

### MMR/PD-L1 protein expression in breast cancers from MMR variant carriers

**Fig. 2:**
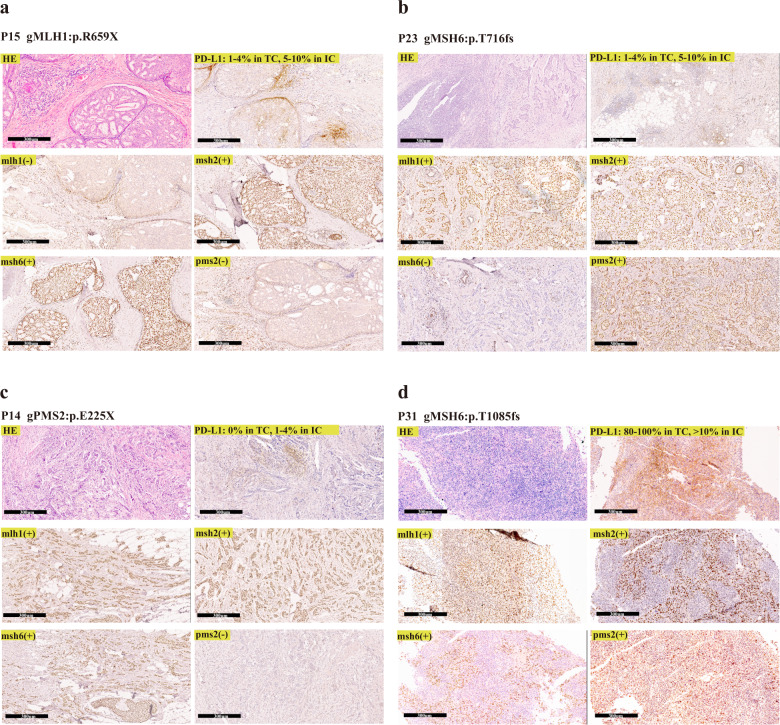
H&E, PD-L1 protein and MMR protein immunostaining of breast cancers from MMR variant carriers. **a** MLH1 variant carrier, mlh1 and pms2 protein loss; **b** MSH6 variant carrier, msh6 protein loss; **c** PMS2 variant carrier, pms2 protein loss; **d** MSH6 variant carrier, strong pd-l1 protein expression. All scale bars = 300 µm.

MMR/PD-L1 protein immunostaining was performed on 13 of the 15 breast cancers carrying MMR pathogenic germline variants. Among them, 5 cancers (5/13, 38.5%) showed total loss of at least one MMR protein (Table 4). The losses affected mlh1 and pms2 in one MLH1 variant carrier (P15) (Fig. 2a), msh6 only in two MSH6 variant carriers (P22, P23) (Fig. 2b), and pms2 only in two PMS2 variant carriers (P14, P20) (Fig. 2c). The protein losses were consistent with the underlying germline variants in MMR genes (Table 4, Fig. 2). In addition, PD-L1 positive expression (>1% in tumor cells (TCs) or immune cells (ICs)) was observed in 9 of the 13 MMR variant carriers (9/13, 69.2%) (Table 4, Fig. 2).

**Table 4:** MMR/PD-L1 protein expression and TMB of breast cancers with pathogenic germline variants in MMR genes.

Case ID	Germline	MMR proteins	MSI	TMB	PD-L1 expression in TC	PD-L1 expression in IC
P12	PMS2:p.R134X	all positive	NA	3.1	1–4%	1–4%
P14	PMS2:p.E225X	pms2-	NA	1.9	0%	1–4%
P20	PMS2:p.R315X	pms2-	NA	5.6	0%	0%
P25	PMS2:p.L351X	all positive	NA	1.3	0%	0%
P26	PMS2:p.V754fs	all positive	NA	0.6	0%	1–4%
P27	PMS2:p.R151fs	NA	NA	NA	NA	NA
P5	MSH6:p.R495X	all positive	NA	0.6	0%	0%
P8	MSH6:p.Y994X	all positive	MSS	3.1	1–4%	>10%
P22	MSH6:p.R922X	msh6-	MSI-L	2.5	0%	0%
P23	MSH6:p.T716fs	msh6-	NA	19.4	1–4%	>10%
P31	MSH6:p.T1085fs	all positive	NA	NA	80–100%	>10%
P2	MSH2:p.Q4X	all positive	NA	NA	1–4%	1–4%
P19	MSH2:p.R877fs	NA	NA	NA	NA	NA
P21	MSH2:p.Q314fs	all positive	MSS	2.5	0%	10%
P15	MLH1:p.R659X	mlh1-, pms2-	NA	15.6	1–4%	5–10%

*MMR* mismatch repair, *TMB* tumor mutation burden, *MSI-L* low microsatellite instability, *MSS* microsatellite stability, *PD-L1* Programmed cell death ligand 1, *IC* immune cell, *TC* tumor cell, *NA* not accessible.

### Somatic mutation profile, TMB and MSI status of breast cancers from MMR variant carriers

Ten of the 15 MMR variant carriers had FFPE tissues with enough tumor purity (>20%) for DNA extraction and depth sequencing on 654 cancer-related genes. To ensure the accuracy of sequencing of FFPE tissue, target region sequencing was performed on FFPE tumor tissues and fresh-frozen tumor tissues from two MMR variant carriers (P8 and P22). Similar somatic mutation profiles (exactly the same oncogenic mutations) and somatic copy number changes were detected in FFPE tumor tissues and fresh-frozen tumor tissues from the same case (Supplementary Table 1). The TMB estimated from FFPE tumor tissues was slightly lower than that from fresh-frozen tumor tissues (Supplementary Table 1).

**Fig. 3:**
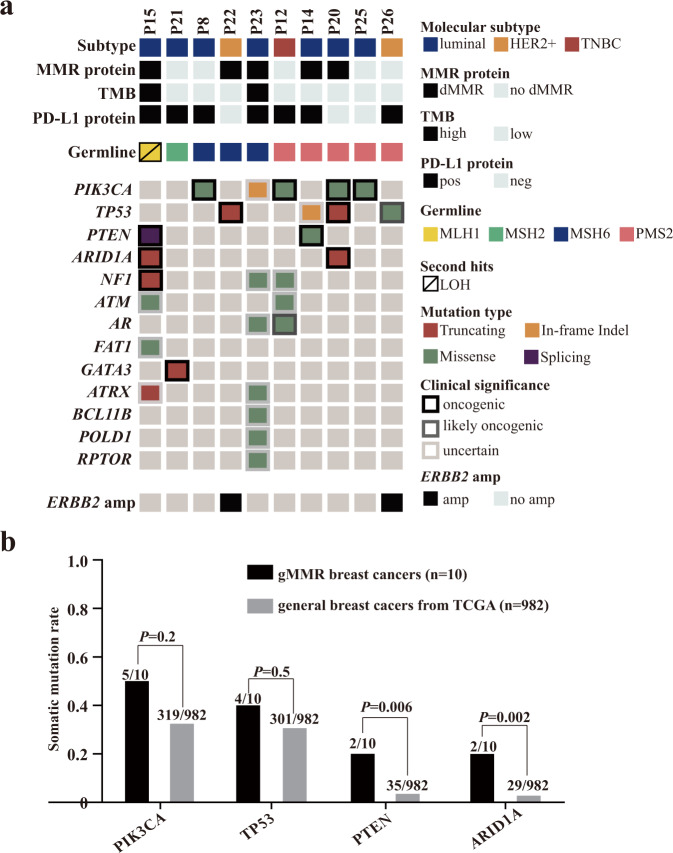
Somatic mutation profiles of breast cancers from MMR variant carriers. **a** Landscape of frequently mutated genes in breast cancers from MMR variant carriers. **b** Comparison of the somatic mutation rates of PIK3CA, TP53, PTEN, and ARID1A between breast cancers carrying MMR germline variants and general breast cancers. Data are analyzed for statistical significance using Fisher’s exact test. Two-sided *P* values < 0.05 were considered to be statistically significant.

Among the 10 breast cancers from MMR variant carriers, PIK3CA (5/10), TP53 (4/10), PTEN (2/10), and ARID1A (2/10) were the most frequently mutated oncogenes or tumor suppressor genes (Fig. 3a). Compared with the somatic mutation profile of general breast cancers reported in the TCGA dataset, breast cancers from MMR variant carriers might have more PTEN mutations [20.0% (2/10) vs. 3.6% (35/982), *P* = 0.006] and ARID1A mutations [20.0% (2/10) vs. 3.0% (29/982), *P* = 0.002] (Fig. 3b). More importantly, 2 of the 10 MMR variant carriers showed TMB-high (>10 Mut/Mb). Second hit events were detected in 1 of the 10 MMR variant carriers (P15: MLH1 carrier, LOH of the wild-type MLH1 allele), and this tumor also exhibited TMB-high and positive PD-L1 expression (Fig. 3a). MSI was estimated in three MMR carriers; only P22 (msh6 protein loss) showed MSI-L, and the other two patients (no MMR protein loss) showed MSS (Table 4).

## Discussion

In this study, 0.19% of the Chinese breast cancer patients were found to carry a pathogenic germline variant in the four MMR genes. Compared with non-carriers, MMR variant carriers showed distinct histology and poor survival. In addition, 5 of the 13 breast cancers from MMR variant carriers showed MMR protein loss consistent with the underlying germline variants, and the breast cancers with germline MMR variants showed a relatively high rate of TMB-high (2/10) and PD-L1 positive expression (9/13).

We found that pathogenic germline variants in MMR genes were rare in consecutive breast cancers (15/8085, 0.19%), and germline variants were more common in PMS2/MSH6 genes than in MLH1/MSH2 genes in breast cancers. In addition, we found that MMR germline variants might affect the tumor phenotype and somatic mutation profile. Our study revealed a potential association between MMR germline variants and medullary/papillary histology in breast cancer, which was also reported in breast cancers from HNPCC families in previous studies^16,17,19^. In addition, PTEN somatic mutations were more common in breast cancers from MMR variant carriers than in those from non-carriers. A similar observation was also reported in endometrioid cancers, in which PTEN loss/somatic mutation were closely associated with MMR deficiency^22,23^. In contrast, the effect of MMR germline variants on the survival of breast cancer was different from that of colorectal cancer. MMR germline variants predict a better survival in colorectal cancers^2^, while our study and another recent study both found that breast cancer patients carrying MMR germline variants might have worse survival than non-carriers^24^. Nevertheless, independent breast cancer cohorts are needed to validate the association between MMR germline variants and breast tumor phenotype because of the limited number of samples in our study.

The poor prognosis of breast cancer patients carrying MMR germline variants urged us to explore whether these patients could benefit from immunotherapy. In a previous clinical trial^7^, MMR protein loss was established as a biomarker predicting the response to immunotherapy, regardless of the cancer type. A recent study also reported that one metastatic breast cancer patient with an MMR germline variant achieved a robust and durable response upon immunotherapy^25^. In this study, we found that one-third of breast cancers (5/13, 38.5%) with MMR germline variants showed MMR protein loss. This rate was similar to the rate reported by a recent study^25^, in which they identified 13 breast cancer patients with MMR germline variants from individuals receiving genetic testing, and found that 42% of them (5/12) displayed MMR protein loss. However, the rate of MMR protein loss in this study was lower than the rate reported in breast cancers from MMR variant carriers with an HNPCC family history (51–71%)^17,18,26^. This might be explained by that the MMR variant carriers in our study were identified from unselected breast cancers. It is well established that TMB and PD-L1 expression are also biomarkers predicting the response to immunotherapy in breast cancers or other solid cancers^27–31^. The TMB-high rate in MMR variant carriers in this study (20.0%) was much higher than that in general breast cancers from the TCGA dataset (1.8%). In addition, the rate of PD-L1 positive expression in MMR variant carriers in this study (69.2%) was much higher than the rate in general breast cancers reported in a previous study (23.6%)^32^. More importantly, we found that the pattern and strength of PD-L1 expression in MMR variant carriers might be different from that in general breast cancer patients. On the one hand, a previous study showed that PD-L1 is usually expressed in ICs instead of TCs in general breast cancers^33^, while 46.2% (6/13) of MMR variant carriers in this study showed positive PD-L1 expression in TCs. On the other hand, the percentage of positive cells expressing PD-L1 in a breast cancer was usually lower than 30% in previous studies^32,34^, while one MMR variant carrier (P31) in this study showed very strong PD-L1 expression (>80% in TCs). In summary, at least 11 of the 15 breast cancers from MMR variant carriers showed MMR protein loss, TMB-high or positive PD-L1 expression, suggesting that the majority of breast cancers from MMR variant carriers might benefit from immunotherapy.

There are several limitations in this study. First, although we have screened MMR germline variants in a large series of breast cancers, the number of MMR variant carriers was very small. Further independent breast cancer cohorts in the further are needed to validate our results. Second, a small portion of breast cancers from MMR variant carriers had genomic DNA with enough quality for MSI detection. Third, MMR variant carriers were associated with poor survival in this this study, and caution is needed when interpreting of these results due to small sample size.

In conclusion, 0.19% of the Chinese breast cancer patients carried a pathogenic germline variant in MMR genes, and MMR variant carriers showed poor survival compared with non-carriers. Breast cancers with germline MMR variants showed relatively high rates of MMR protein loss, TMB-high and PD-L1 positive expression compared with general breast cancers, suggesting that some breast cancer patients with germline MMR variants might benefit from immunotherapy.

## Methods

### Patients

A total of 8085 consecutive breast cancer patients who were treated at the Breast Center of Peking University Cancer Hospital from October 2003 to May 2015 were included in this study^35^. The cohort was unselected for age at diagnosis and family history. Detailed demographic information and tumor characteristics of each patient were collected from medical records and/or telephone interviews. Estrogen receptor (ER), progesterone receptor (PR), and human epidermal growth factor receptor 2 (HER2) status were determined using the breast tumor tissue obtained from a core needle biopsy or taken from surgery. ER or PR immunostaining was considered positive when >1% of the TCs showed positive nuclear staining. HER2 positivity was defined as a score of 3+ via immunohistochemical staining or HER2 gene amplification via fluorescence in situ hybridization. Informed written consent was obtained from all participants. This study was approved by the Research and Ethics Committee of Peking University Cancer Hospital.

### MMR germline variant classification

Panel sequencing (including four MMR genes: MLH1, MSH2, MSH6, and PMS2) was performed on genomic DNA extracted from the peripheral blood of the 8085 unselected breast cancer patients^35^. In this study, we reanalyzed the MMR germline variants detected in our previous report. Germline variations were called with GATK (version 3.6). Annotations were defined using ANNOVAR. Only variants with <1% population frequency in the population databases including gnomAD (v3.1.2) and TOPMed (version 20210514) were collected (Supplementary Data 1). Among these, truncating variants (nonsense and frameshift variants) were included in this study, but truncating variants in the last 55 base pairs of the penultimate exon or last exon that potentially avoid nonsense-mediated messenger RNA decay and do not influence known functional domains were excluded. For splice-site, synonymous, nonsynonymous, in-frame, and stop-loss variants, only variants classified as pathogenic or likely pathogenic by ClinVar (version 20210501) were included in the analysis. Variants with conflicting interpretations of pathogenicity in ClinVar were further annotated according to the ACMG/AMP standards and guidelines^36^, with supporting data from function prediction software, public literature, and curated databases. Variants classified as pathogenic or likely pathogenic were considered as pathogenic in this study.

### H&E staining

Hematoxylin and eosin (H&E) staining was performed on 4 μm sections of formalin-fixed, paraffin-embedded breast cancer tissue from each MMR variant carrier. The staining procedures were as follows: dewaxing, dehydration, hematoxylin, differentiation, bluing, eosin, dehydration, clearing, and cover-slipping. The pathologists histopathologically evaluated the tumor cell area in each H&E section.

### Immunohistochemistry (IHC)

Immunohistochemistry was performed on 4 μm sections of formalin-fixed, paraffin-embedded tissue from MMR variant carriers. MMR protein detection was carried out using primary antibodies against mlh1 (clone GM002, mouse monoclonal antibody, catalogue numbers: GT230407, working solution, Gene Tech), msh2 (clone RED2, rabbit monoclonal antibody, catalogue numbers: GT231007, working solution, Gene Tech), msh6 (clone EP49, rabbit monoclonal antibody, catalogue numbers: GT219507, working solution, Gene Tech), and pms2 (clone EP51, rabbit monoclonal antibody, catalogue numbers: GT215907, working solution, Gene Tech). MMR protein loss was defined as nuclear immunostaining negative for one or several MMR proteins in the TCs on whole section slides. PD-L1 IHC staining was performed using the primary antibody for the pd-l1 protein (clone SP142, rabbit monoclonal antibody, catalogue numbers: ab228462, dilution ratio 1:400, Abcam). The percentage of PD-L1 was scored as a continuous variable. The threshold for PD-L1 positivity was set at ≥1% positive TCs and/or ICs. At least one positive control (embryo tissue section) and one negative control (PBS instead of primary antibody) were used in each IHC assay (Supplementary Fig. 1). The tumor sections were independently scored for PD-L1 expression in TCs/ICs blinded to the germline status, MMR protein status and clinical data.

### Target region sequencing on breast cancers from MMR variant carriers

Ten breast cancers with germline variants in MMR genes had enough TCs in the formalin-fixed and paraffin-embedded (FFPE) blocks for target region sequencing. For each block, a H&E stained slide was reviewed by a pathologist to ensure that at least 20% of the nucleated cells in the slide were derived from the TCs. Genomic DNA was extracted from FFPE sections by using GeneRead DNA FFPE Kit (QIAGEN, Germany). For each sample, 100–300 ng of DNA was prepared to construct a paired-end DNA library. The DNA was subjected to 654 cancer-related genes (including the 4 MMR genes) target capture by using the Solid Tumor Comprehensive Test Kit. The products were sequenced on an Illumina HiSeq 4000 sequencing platform. The tumor samples were sequenced at an average depth of 570× on the target region. Matched blood samples were sequenced at an average depth of 1400× in the same regions to identify and filter germline variants. In addition, fresh-frozen tumor tissues were available for 2 of the 10 MMR variant carriers (P8 and P22). We extracted DNA from the two fresh-frozen tumor samples by using the DNeasy Blood & Tissue Kit (QIAGEN, Germany), which were also subjected to 654 cancer-related genes target sequencing with at an average depth of 1576× on the target region.

Sequenced reads were aligned to the human reference genome (NCBI Build 37) by the Burrows-Wheeler Aligner (version 0.1.22). Somatic indels and single nucleotide variations were called by MutLoc with an additional filter to exclude artificial mutations introduced by FFPE tissue. In brief, duplicates and soft clipped reads removed data was analyzed in MutLoc with these parameters (align quality: 30; strand bias: 0.05; keep the mutation site with highest align quality if more than one mutation sites were examined within 11 bp; keep the mutation sites supported by at least three different reads). In addition, we filtered out single strand bias based on a read pair orientation of larger than 20:1. Somatic copy number variations were called by GATK (version 3.6). Function annotations were defined using ANNOVAR. All the somatic mutations detected in breast cancers from the ten MMR variant carriers were listed in Supplementary Data 2. Tumor mutation burden (TMB) was defined as the number of non-synonymous somatic mutations (single nucleotide variants and small insertions/deletions) per mega-base in coding regions. The TMB of each tumor was determined on 1.6 Mb of sequenced DNA and reported as mutations/Mb. TMB ≥ 10 Mut/Mb was considered as TMB-high.

### PCR-based MSI detection

Genomic DNA extracted from the FFPE sections of one case and fresh-frozen tumor tissues of two cases had sufficient quality for MSI detection. Genomic DNA extracted from matched blood samples served as controls to filter germline variants in microsatellite loci. Five mononucleotide loci (NR-21, BAT-26, BAT-25, NR-24, MONO-27) were used for MSI analysis (Promega, USA). Tumors were classified MSI-H (≥2 loci exhibit instability), MSI-L (only one locus exhibits instability) or microsatellite stable (MSS, all five loci exhibit stability).

### The Cancer Genome Atlas public dataset analysis

The somatic mutation data of 982 breast cancers were downloaded from the TCGA database (https://portal.gdc. cancer.gov/projects). The TMB of each tumor was determined on 30 Mb of sequenced DNA and reported as mutations/Mb. TMB ≥ 10 Mut/Mb was considered as TMB-high.

### Statistical analysis

Categorical variables were compared using the *χ*2 test or Fisher’s exact test, where appropriate. Continuous variables were tested with a *t*-test, where appropriate. RFS was defined as from the time of diagnosis to first recurrence (local or distant), or death from breast cancer (for patients without a recorded relapse) or date of last follow-up. DRFS was defined as from the time of diagnosis to first distant recurrence, or death from breast cancer (for patients without a record of recurrence), or the date of the last follow up. Survival was estimated using the Kaplan–Meier method. Univariate and multivariate Cox proportional hazards models were used to determine whether a factor was associated with survival. Two-sided *P* values < 0.05 were considered to be statistically significant. All analyses were performed using SPSS 20.0 software.

### Reporting summary

Further information on research design is available in the Nature Research Reporting Summary linked to this article.

## Supplementary information

Supplementary figure and table

Dataset 1

Dataset 2

Reporting Summary Checklist

## Data Availability

The next-generation sequencing data analyzed in this study have been uploaded as Supplementary data. The other relevant data are available from the authors upon reasonable request.

## References

[1] Hendriks YMC (2006). Diagnostic Approach and Management of Lynch Syndrome (Hereditary Nonpolyposis Colorectal Carcinoma): a Guide for Clinicians. Ca Cancer J. Clin..

[2] Gryfe R (2000). Tumor Microsatellite Instability and Clinical Outcome in Young Patients with Colorectal Cancer. N. Engl. J. Med..

[3] Boland CR, Goel A (2010). Microsatellite Instability in Colorectal Cancer. Gastroenterology.

[4] Prolla TA (1998). Tumour susceptibility and spontaneous mutation in mice deficient in Mlh1, Pms1 and Pms2 DMA mismatch repair. Nat. Genet..

[5] Le DT (2015). PD-1 Blockade in Tumors with Mismatch-Repair Deficiency.. N Engl J. Med..

[6] Overman MJ (2017). Nivolumab in patients with DNA mismatch repair deficient/microsatellite instability high metastatic colorectal cancer: Update from CheckMate 142. J. Clin. Oncol..

[7] Le DT (2017). Mismatch repair deficiency predicts response of solid tumors to PD-1 blockade. Science.

[8] Müller A (2002). Exclusion of breast cancer as an integral tumor of hereditary nonpolyposis colorectal cancer. Cancer Res..

[9] Broeke SWten (2015). Lynch Syndrome Caused by Germline PMS2 mutations: delineating the cancer risk. J. Clin. Oncol..

[10] Win AK (2012). Colorectal and Other Cancer Risks for Carriers and Noncarriers From Families With a DNA Mismatch Repair Gene Mutation: A Prospective Cohort Study. J. Clin. Oncol..

[11] Engel C (2012). Risks of Less Common Cancers in Proven Mutation Carriers With Lynch Syndrome. J. Clin. Oncol..

[12] Goldberg M (2017). Association between the Lynch syndrome gene MSH2 and breast cancer susceptibility in a Canadian familial cancer registry. J. Med Genet..

[13] Roberts ME (2018). MSH6 and PMS2 germ-line pathogenic variants implicated in Lynch syndrome are associated with breast cancer. Genet. Med. Off. J. Am. Coll. Med. Genet..

[14] Harkness EF (2015). Lynch syndrome caused by MLH1 mutations is associated with an increased risk of breast cancer: a cohort study. J. Med. Genet..

[15] Rashid MU (2016). A novel deleterious c.2656G>T MSH2 germline mutation in a Pakistani family with a phenotypic overlap of hereditary breast and ovarian cancer and Lynch syndrome. Hered. Cancer Clin. Pr..

[16] Jensen UB (2009). Mismatch repair defective breast cancer in the hereditary nonpolyposis colorectal cancer syndrome. Breast Cancer Res. Tr..

[17] Walsh MD (2010). Lynch syndrome-associated breast cancers: clinicopathologic characteristics of a case series from the colon cancer family registry. Clin. Cancer Res..

[18] Buerki N (2011). Evidence for breast cancer as an integral part of lynch syndrome. Genes Chromosom. Cancer.

[19] Kanaya N (2018). Clinicopathological features of breast cancer in Japanese female patients with Lynch syndrome. Breast Cancer-tokyo.

[20] Sorscher S (2019). Rationale for evaluating breast cancers of Lynch syndrome patients for mismatch repair gene expression. Breast Cancer Res. Tr..

[21] Porkka NK (2020). Does breast carcinoma belong to the Lynch syndrome tumor spectrum? - Somatic mutational profiles vs. ovarian and colorectal carcinomas. Oncotarget.

[22] Djordjevic B, Barkoh BA, Luthra R, Broaddus RR (2013). Relationship between PTEN, DNA mismatch repair, and tumor histotype in endometrial carcinoma: retained positive expression of PTEN preferentially identifies sporadic non-endometrioid carcinomas. Mod. Pathol..

[23] Levine DA (2013). Integrated genomic characterization of endometrial carcinoma. Nature.

[24] Cheng AS (2019). Mismatch repair protein loss in breast cancer: clinicopathological associations in a large British Columbia cohort. Breast Cancer Res. Tr..

[25] Schwartz CJ (2021). Morphological and genomic characteristics of breast cancers occurring in individuals with Lynch Syndrome. Clin. Cancer Res. Clincanres.

[26] Win AK, Lindor NM, Jenkins MA (2013). Risk of breast cancer in Lynch syndrome: a systematic review. Breast Cancer Res..

[27] Kim ST (2018). Comprehensive molecular characterization of clinical responses to PD-1 inhibition in metastatic gastric cancer. Nat. Med..

[28] Rizvi NA (2015). Cancer immunology. Mutational landscape determines sensitivity to PD-1 blockade in non-small cell lung cancer. Sci. N. Y.

[29] Huang J (2018). Safety, activity and biomarkers of SHR-1210, an anti-PD-1 antibody, for patients with advanced esophageal carcinoma. Clin. Cancer Res. 24, Clincanres.

[30] Schmid P (2019). Atezolizumab plus nab-paclitaxel as first-line treatment for unresectable, locally advanced or metastatic triple-negative breast cancer (IMpassion130): updated efficacy results from a randomised, double-blind, placebo-controlled, phase 3 trial. Lancet Oncol..

[31] Schmid P (2018). Atezolizumab and Nab-Paclitaxel in Advanced Triple-Negative Breast Cancer. New Engl J. Med.

[32] Buisseret L (2016). Tumor-infiltrating lymphocyte composition, organization and PD-1/ PD-L1 expression are linked in breast cancer. Oncoimmunology.

[33] Cimino-Mathews A (2015). PD-L1 (B7-H1) expression and the immune tumor microenvironment in primary and metastatic breast carcinomas. Hum. Pathol..

[34] Solinas C (2019). BRCA gene mutations do not shape the extent and organization of tumor infiltrating lymphocytes in triple negative breast cancer. Cancer Lett..

[35] Sun J (2017). Germline Mutations in Cancer Susceptibility Genes in a Large Series of Unselected Breast Cancer Patients. Clin. Cancer Res..

[36] Richards S (2015). Standards and guidelines for the interpretation of sequence variants: a joint consensus recommendation of the American College of Medical Genetics and Genomics and the Association for Molecular Pathology. Genet. Med..

