# Supplementary material for: Clinical relevance of pathogenic germline variants in mismatch repair genes in Chinese breast cancer patients
Source: NPJ Breast Cancer. 2022 Apr 21;8:52. doi: 10.1038/s41523-022-00417-x (PMC9023502; doi:10.1038/s41523-022-00417-x)
Supplement: Supplementary file 1 — Supplementary figure and table [file 41523_2022_417_MOESM1_ESM.pdf]

Supplementary Table 1. Comparison of somatic mutation profiles detected from FFPE tissue and that detected from fresh-frozen tissue.

Case ID	Tumor tissue type	Somatic mutations	Somatic CNVs	TMB (/Mb)
P8	FFPE	CDC73 p.W492* (pathogenic); PIK3CA p.H1047L (pathogenic); SETD3 p.T226N (Uncertain significance); AMER1 p.G149P (Uncertain significance); BACH2 p.D223A (Uncertain significance).	None	3.1
P8	Frozen-fresh	CDC73 p.W492* (pathogenic); PIK3CA p.H1047L (pathogenic); SETD3 p.T226N (Uncertain significance); PTPRD p.K135N (Uncertain significance); TLR4 p.L258F (Uncertain significance).	None	3.8
P22	FFPE	TP53 p.N131Tfs*39 (pathogenic); PHF6 p.H86Ifs*11 (pathogenic); FANCG p.R423H (Uncertain significance); MED12 p.R1205H (Uncertain significance).	ERBB2 amp (30 copy)	2.5
P22	Frozen-fresh	TP53 p.N131Tfs*39 (pathogenic); PHF6 p.H86Ifs*11 (pathogenic); FANCG p.R423H (Uncertain significance); MED12 p.R1205H (Uncertain significance); LIFR p.N169S (Uncertain significance).	ERBB2 amp (34 copy)	4.4

Abbreviations: FFPE, formalin-fixed and paraffin-embedded tissues; CNV, copy number variants; TMB, tumor mutation burden.

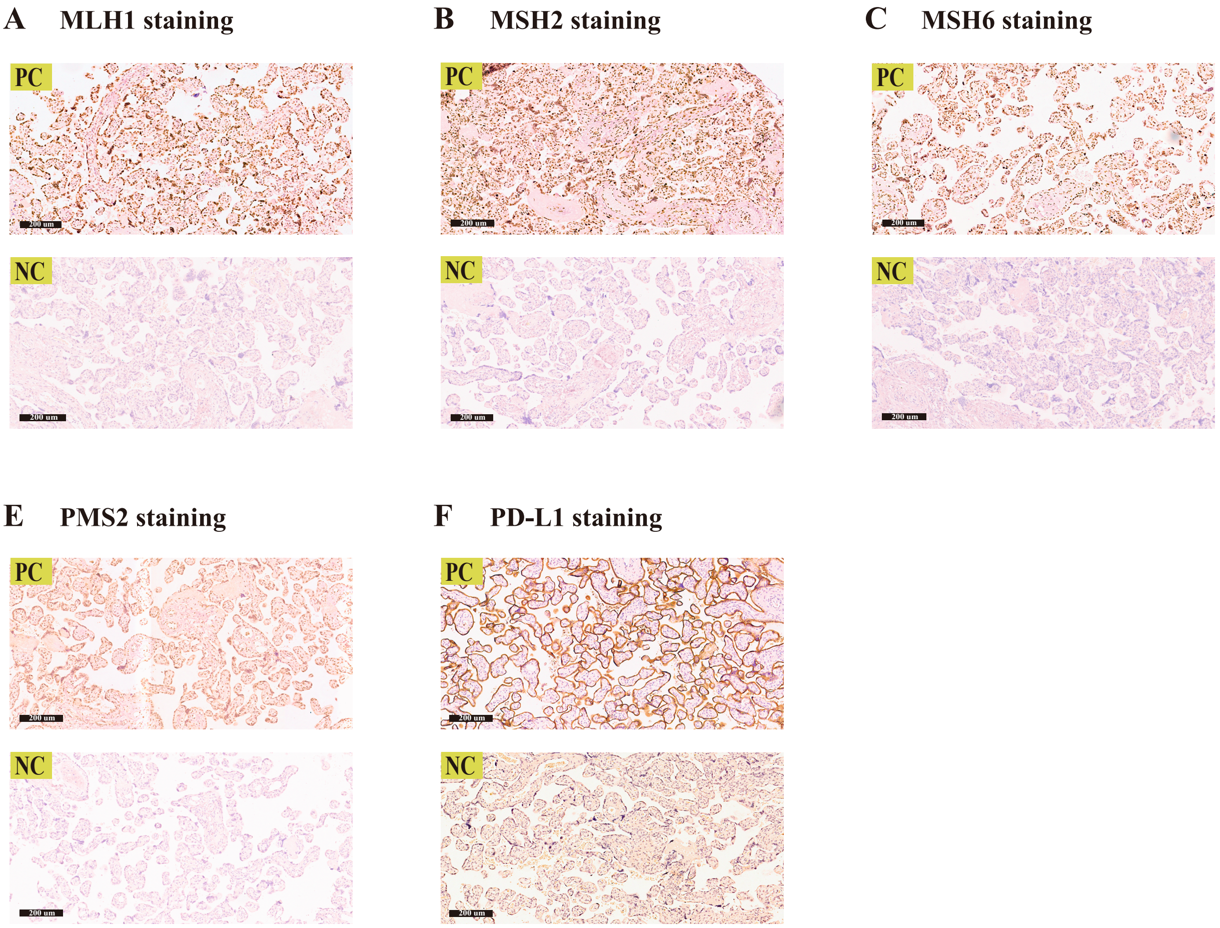

Supplementary Figure 1. The positive control (PC) and negative control (NC) for MMR/PD-L1 protein immunohistochemistry assays. All scale bars=200 um
