# Supplementary material for: Clinical relevance of pathogenic germline variants in mismatch repair genes in Chinese breast cancer patients
Source: NPJ Breast Cancer. 2022 Apr 21;8:52. doi: 10.1038/s41523-022-00417-x (PMC9023502; doi:10.1038/s41523-022-00417-x)
Supplement: Supplementary file 4 — Reporting Summary Checklist [file 41523_2022_417_MOESM4_ESM.pdf]

nature portfolio

Corresponding author(s): Yuntao Xie

Last updated by author(s): Mar 5, 2022

Reporting Summary

Nature Portfolio wishes to improve the reproducibility of the work that we publish. This form provides structure for consistency and transparency in reporting. For further information on Nature Portfolio policies, see our Editorial Policies and the Editorial Policy Checklist.

Statistics

For all statistical analyses, confirm that the following items are present in the figure legend, table legend, main text, or Methods section.

n/a	Confirmed	
☐	☒	The exact sample size (*n*) for each experimental group/condition, given as a discrete number and unit of measurement
☐	☒	A statement on whether measurements were taken from distinct samples or whether the same sample was measured repeatedly
☐	☒	The statistical test(s) used AND whether they are one- or two-sided<br>*Only common tests should be described solely by name; describe more complex techniques in the Methods section.*
☒	☐	A description of all covariates tested
☒	☐	A description of any assumptions or corrections, such as tests of normality and adjustment for multiple comparisons
☒	☐	A full description of the statistical parameters including central tendency (e.g. means) or other basic estimates (e.g. regression coefficient) AND variation (e.g. standard deviation) or associated estimates of uncertainty (e.g. confidence intervals)
☒	☐	For null hypothesis testing, the test statistic (e.g. *F*, *t*, *r*) with confidence intervals, effect sizes, degrees of freedom and *P* value noted<br>*Give P values as exact values whenever suitable.*
☒	☐	For Bayesian analysis, information on the choice of priors and Markov chain Monte Carlo settings
☒	☐	For hierarchical and complex designs, identification of the appropriate level for tests and full reporting of outcomes
☒	☐	Estimates of effect sizes (e.g. Cohen's *d*, Pearson's *r*), indicating how they were calculated

Our web collection on statistics for biologists contains articles on many of the points above.

Software and code

Policy information about availability of computer code

Data collection

A total of 8085 consecutive breast cancer patients who were treated at the Breast Center of Peking University Cancer Hospital from October 2003 to May 2015 were included in this study. The cohort was unselected for age at diagnosis and family history. Detailed demographic information and tumor characteristics of each patient were collected from medical records and/or telephone interviews. Panel sequencing (including four MMR genes: MLH1, MSH2, MSH6, and PMS2) was performed on genomic DNA extracted from the peripheral blood of the 8085 unselected breast cancer patients. Ten breast cancers with germline variants in MMR genes had enough tumor cells in the formalin-fixed and paraffin-embedded (FFPE) blocks for target region sequencing. For each block, a hematoxylin and eosin (H&E) stained slide was reviewed by a pathologist to ensure that at least 20% of the nucleated cells in the slide were derived from the tumor cells. Genomic DNA was extracted from FFPE sections by using GeneRead DNA FFPE Kit (QIAGEN, Germany). For each sample, 100-300 ng of DNA was prepared to construct a paired-end DNA library. The DNA was subjected to 654 cancer-related genes (including the 4 MMR genes) target capture by using the Solid Tumor Comprehensive Test Kit. The products were sequenced on an Illumina HiSeq 4000 sequencing platform. The tumor samples were sequenced at an average depth of 570× on the target region. Matched blood samples were sequenced at an average depth of 1400× in the same regions to identify and filter germline variants. In addition, fresh-frozen tumor tissues were available for 2 of the 10 MMR variant carriers (P8 and P22). We extracted DNA from the two fresh-frozen tumor samples by using the DNeasy Blood & Tissue Kit (QIAGEN, Germany), which were also subjected to 654 cancer-related genes target sequencing with at an average depth of 1576× on the target region.

Data analysis

MMR germline variant classification
Panel sequencing (including four MMR genes: MLH1, MSH2, MSH6, and PMS2) was performed on genomic DNA extracted from the peripheral blood of the 8085 unselected breast cancer patients. In this study, we reanalyzed the MMR germline variants detected in our previous report. Germline variations were called with GATK (version 3.6). Annotations were defined using ANNOVAR. Only variants with <1% population frequency in the population databases including gnomAD (v3.1.2) and TOPMed (version 20210514) were collected (Supplementary Data 1). Among these, truncating variants (nonsense and frameshift variants) were included in this study, but truncating variants in the last 55 base pairs of the penultimate exon or last exon that potentially avoid nonsense-mediated messenger RNA decay and do not influence known functional domains were excluded. For splice-site, synonymous, nonsynonymous, in-frame, and stop-loss variants, only variants classified as pathogenic or likely pathogenic by ClinVar (version 20210501) were included in the analysis. Variants with conflicting interpretations of

pathogenicity in ClinVar were further annotated according to the ACMG/AMP standards and guidelines35, with supporting data from function prediction software, public literature, and curated databases. Variants classified as pathogenic or likely pathogenic were considered as pathogenic in this study.
Target region sequencing on breast cancers from MMR variant carriers
Sequenced reads were aligned to the human reference genome (NCBI Build 37) by the Burrows-Wheeler Aligner (version 0.1.22). Somatic indels and single nucleotide variations (SNVs) were called by MutLoc with an additional filter to exclude artificial mutations introduced by FFPE tissue. In brief, duplicates and soft clipped reads removed data was analyzed in MutLoc with these parameters (align quality: 30; strand bias: 0.05; keep the mutation site with highest align quality if more than one mutation sites were examined within 11 bp; keep the mutation sites supported by at least three different reads). In addition, we filtered out single strand bias based on a read pair orientation of larger than 20:1. Somatic copy number variations (CNVs) were called by GATK (version 3.6). Function annotations were defined using ANNOVAR. All the somatic mutations detected in breast cancers from the ten MMR variant carriers were listed in Supplementary Data 2. Tumor mutation burden (TMB) was defined as the number of non-synonymous somatic mutations (single nucleotide variants and small insertions/deletions) per mega-base in coding regions. The TMB of each tumor was determined on 1.6 Mb of sequenced DNA and reported as mutations/Mb. TMB≥10 Mut/Mb was considered as TMB-high.
Statistical analysis
Categorical variables were compared using the χ^2 test or Fisher's exact test, where appropriate. Continuous variables were tested with a t test, where appropriate. RFS was defined as from the time of diagnosis to first recurrence (local or distant), or death from breast cancer (for patients without a recorded relapse) or date of last follow-up. DRFS was defined as from the time of diagnosis to first distant recurrence, or death from breast cancer (for patients without a record of recurrence), or the date of the last follow up. Survival was estimated using the Kaplan-Meier method. Univariate and multivariate Cox proportional hazards models were used to determine whether a factor was associated with survival. Two-sided P values less than 0.05 were considered to be statistically significant. All analyses were performed using SPSS 20.0 software.

For manuscripts utilizing custom algorithms or software that are central to the research but not yet described in published literature, software must be made available to editors and reviewers. We strongly encourage code deposition in a community repository (e.g. GitHub). See the Nature Portfolio guidelines for submitting code & software for further information.

Data

Policy information about availability of data

All manuscripts must include a data availability statement. This statement should provide the following information, where applicable:

- Accession codes, unique identifiers, or web links for publicly available datasets
- A description of any restrictions on data availability
- For clinical datasets or third party data, please ensure that the statement adheres to our policy

The next-generation sequencing data analyzed in this study have been uploaded as Supplementary data. The other relevant data are available from the authors upon reasonable request.

Field-specific reporting

Please select the one below that is the best fit for your research. If you are not sure, read the appropriate sections before making your selection.

☒ Life sciences ☐ Behavioural & social sciences ☐ Ecological, evolutionary & environmental sciences

For a reference copy of the document with all sections, see nature.com/documents/nr-reporting-summary-flat.pdf

Life sciences study design

All studies must disclose on these points even when the disclosure is negative.

Sample size	A total of 8085 consecutive breast cancer patients who were treated at the Breast Center of Peking University Cancer Hospital from October 2003 to May 2015.
Data exclusions	N/A
Replication	N/A
Randomization	N/A
Blinding	N/A

Reporting for specific materials, systems and methods

We require information from authors about some types of materials, experimental systems and methods used in many studies. Here, indicate whether each material, system or method listed is relevant to your study. If you are not sure if a list item applies to your research, read the appropriate section before selecting a response.

Materials & experimental systems

n/a	Involved in the study
☐	☒ Antibodies
☒	☐ Eukaryotic cell lines
☒	☐ Palaeontology and archaeology
☒	☐ Animals and other organisms
☐	☒ Human research participants
☒	☐ Clinical data
☒	☐ Dual use research of concern

Methods

n/a	Involved in the study
☒	☐ ChIP-seq
☒	☐ Flow cytometry
☒	☐ MRI-based neuroimaging

Antibodies

Antibodies used

primary antibodies for mlh1 (clone GM002, mouse monoclonal antibody, catalogue numbers: GT230407, working solution, Gene Tech), msh2 (clone RED2, rabbit monoclonal antibody, catalogue numbers: GT231007, working solution, Gene Tech), msh6 (clone EP49, rabbit monoclonal antibody, catalogue numbers: GT219507, working solution, Gene Tech), and pms2 (clone EP51, rabbit monoclonal antibody, catalogue numbers: GT215907, working solution, Gene Tech).primary antibody for pd-l1 protein (clone SP142, rabbit monoclonal antibody, catalogue numbers: ab228462, dilution ratio 1:400, Abcam).

Validation

In each IHC assay in this study, we use human embryonic tissue as a positive control for primary antibodies, and used PBS instead of primary antibodies as negative control. These IHC data are available from the corresponding author upon reasonable request.

Human research participants

Policy information about studies involving human research participants

Population characteristics

A total of 8085 consecutive breast cancer patients who were treated at the Breast Center of Peking University Cancer Hospital from October 2003 to May 2015. The cohort were unselected for age at diagnosis and family history. Panel sequencing (including four MMR genes: MLH1, MSH2, MSH6, and PMS2) were performed on genomic DNA extracted from peripheral blood of the 8085 unselected breast cancer patients, as described in our previous report. Criteria for classifying pathogenic variants as described in our previous report. Variants classified to be pathogenic or likely pathogenic were considered as pathogenic in this study. Detailed demographic information and tumor characteristics of each patient were collected from medical records and/or telephone interviews. Estrogen receptor (ER), progesterone receptor (PR), and human epidermal growth factor receptor 2 (HER2) status were determined using the breast tumor tissue obtained from a core-needle biopsy or taken from surgery, Criteria for defining ER/PR/HER2 positive as described elsewhere were used.

Recruitment

This is a retrospective study. The study population were consecutive breast cancer patients who were treated at the Breast Center of Peking University Cancer Hospital from October 2003 to May 2015. They were unselected for age at diagnosis and family history.

Ethics oversight

This study was approved by the Research and Ethics Committee of Peking University Cancer Hospital.

Note that full information on the approval of the study protocol must also be provided in the manuscript.
